# Posterior Cingulate Lactate as a Metabolic Biomarker in Amnestic Mild Cognitive Impairment

**DOI:** 10.1155/2015/610605

**Published:** 2015-08-31

**Authors:** Kurt E. Weaver, Todd L. Richards, Rebecca G. Logsdon, Ellen L. McGough, Satoshi Minoshima, Elizabeth H. Aylward, Natalia M. Kleinhans, Thomas J. Grabowski, Susan M. McCurry, Linda Teri

**Affiliations:** ^1^Department of Radiology, University of Washington, P.O. Box 357115, Seattle, WA 98195, USA; ^2^Integrated Brain Imaging Center, University of Washington, Seattle, WA 98195, USA; ^3^Department of Psychosocial and Community Health, University of Washington, Seattle, WA 98195, USA; ^4^Department of Rehabilitation Medicine, University of Washington, Seattle, WA 98195, USA; ^5^Center for Integrative Brain Research, Seattle Children's Research Institute, Seattle, WA 98101, USA; ^6^Department of Neurology, University of Washington, Seattle, WA 98195, USA

## Abstract

Mitochondrial dysfunction represents a central factor within the pathogenesis of the Alzheimer's disease (AD) spectrum. We hypothesized that in vivo measurements of lactate (lac), a by-product of glycolysis, would correlate with functional impairment and measures of brain health in a cohort of 15 amnestic mild cognitive impairment (aMCI) individuals. Lac was quantified from the precuneus/posterior cingulate (PPC) using 2-dimensional J-resolved magnetic resonance spectroscopy (MRS). Additionally, standard behavioral and imaging markers of aMCI disease progression were acquired. PPC lac was negatively correlated with performance on the Wechsler logical memory tests and on the minimental state examination even after accounting for gray matter, cerebral spinal fluid volume, and age. No such relationships were observed between lac and performance on nonmemory tests. Significant negative relationships were also noted between PPC lac and hippocampal volume and PPC functional connectivity. Together, these results reveal that aMCI individuals with a greater disease progression have increased concentrations of PPC lac. Because lac is upregulated as a compensatory response to mitochondrial impairment, we propose that J-resolved MRS of lac is a noninvasive, surrogate biomarker of impaired metabolic function and would provide a useful means of tracking mitochondrial function during therapeutic trials targeting brain metabolism.

## 1. Introduction

Effectively all contemporary theories of Alzheimer's disease (AD) pathogenesis integrate impaired brain metabolism into the concert of factors precipitating neurodegeneration and clinical disability. One of the earliest observable metabolic impairments consistently linked with cognitive dysfunction across the AD spectrum is reduced brain glucose metabolism [1]. Studies of [18F] fluorodeoxyglucose uptake using positron emission tomography (FDG-PET) reveal that glucose hypometabolism in amnestic mild cognitive impairment (aMCI), a cohort of individuals at increased risk for transitioning to AD, is associated with poorer memory capacity [2] and reductions in hippocampal volume [3]. At a systems level, FDG-PET hypometabolism in aMCI tends to develop more within regions of the default mode network (DMN) [4]. The precuneus/posterior cingulate (PPC), one of the main integrative hubs of the DMN, is particularly sensitive to reductions in glucose consumption. The PPC is highly interconnected with the hippocampus and the interaction between the two structures plays a critical role in orchestrating episodic memory recall [5, 6]. Combined, these and many other findings have led to the use of FDG-PET as a biomarker for tracking AD-related metabolic impairments and a prediction that FDG-PET will help increase the clinical efficacy of therapeutics targeting brain metabolism abnormalities [7].

However, numerous other well documented metabolic impairments have been noted constituting additional and likely overlapping pathological processes (reviewed in [8, 9]). The general interpretation from an extensive animal and human literature is that prodromal stages of AD, including aMCI, are marked with reduced vascular integrity, compromised mitochondrial function and elevated oxidative stress [1, 10, 11]. As a result, mitochondrial bioenergetics has become a source of therapeutic targets in predemented stages of the spectrum [12, 13].

In analogous clinical scenarios presenting with disrupted mitochondrial function (e.g., mitochondrial disorders such as Leigh syndrome or MELAS [14, 15]), lactate (lac), the by-product of anaerobic glycolysis, is elevated in part as compensation for aerobic metabolic impairment [16–18]. Thus, 1H magnetic resonance spectroscopic (MRS) quantification of lac is often used as a diagnostic marker for inborn errors of metabolism [15, 19] as well as other clinical scenarios affecting basal metabolic function (e.g., hypoxia/ischemia [20]). Taken together, we hypothesize that concentrations of brain lac would serve as a biomarker of metabolic impairment across the aMCI spectrum, potentially as a reflection of mitochondrial dysfunction.

As far as we are aware, only two previous MRS reports have attempted to quantify brain lac in AD. Stoppe et al. [21] reported no differences between patients and controls in lac concentrations extracted from a voxel in the parietal lobe. More critically, Ernst et al. [22] failed altogether to detect a lac signal in AD from either prefrontal or lateral temporal cortex. The lack of signal change or detection within these studies may stem from a variety of concerns such as choice of location (i.e., spectra may have been acquired from cortex showing relative immunity to AD pathology) or perhaps and a more likely scenario as a result of spectral overlap and contamination [23]. Both previous studies relied on a standard “short-TE” spectroscopic approach. This technique which acquires frequency induction decays (FIDs) at a single echo time (typically between 30–35 ms) provides reliable quantification of the classic MRS metabolites including N-acetylaspartate [NAA] but yields limited quantification capacity for metabolites with overlapping resonance frequencies [24]. Within the MR proton spectrum the lac signature exists as a doublet peak located at 1.35 ppm. This peak however is buried within a robust lipid and macromolecule resonance at overlapping frequencies. Therefore using traditional single shot short-TE spectroscopic imaging, a significant rise in concentration (e.g., as seen during mitochondrial disease) is usually necessary to expose the lac doublet [25].

The purpose of this study is to first reexamine whether brain lac could be quantified in vivo using noninvasive imaging in a group aMCI subjects. We focused specifically on the PPC because of the consistent reductions reported across the FDG-PET literature in aMCI (c.f. [26]). To circumvent the known spectral contamination concerns of lac, we capitalized on the J-coupling behavior of the lac molecule. We quantified PPC lac using 2-dimensional J-resolved magnetic resonance spectroscopy (J-rMRS) [27]. 2D J-rMRS enables the separation of J-coupling information from chemical shift by encoding J-coupling in the t2 dimension of a 2D spectrum using an array of echo times [28]. The addition of this second frequency dimension allows for separation of lac from lipids and macromolecules, molecules that lack J-coupling properties. Second, we contrasted PPC lac with behavioral and imaging characteristics that are commonly used as markers of disease progression across the AD spectrum. Specifically, PET studies consistently observe that PPC metabolic impairments in aMCI and AD are significantly associated with memory dysfunction, hippocampal volumetry, and other imaging markers of disease pathology (see [1] for review). By correlating PPC lac with known markers of disease progression, we aim to establish the validity of PPC lac as a valid metabolic biomarker in aMCI.

## 2. Material and Methods

### 2.1. Subjects and Cognitive Screening

Fifteen community-dwelling aMCI individuals (mean age 85.5 yrs. old, 5 males) were included (see Table 1 for demographics). Study recruitment and screening consisted of a semistructured interview, neuropsychological screening tests, and an expert consensus panel to review screening data. Participants were classified as having amnestic MCI based on the Petersen criteria [29] and was determined from a combination of cognitive test scores, screening interview data, and consensus case review to identify persons with memory problems that would be consistent with a clinical subtype of single domain amnestic MCI, including (1) memory complaint, (2) impaired memory for age and education, (3) preserved general cognitive function, (4) essentially preserved activities of daily living, and (5) not already diagnosed with dementia. Specific measures included self-reported memory loss, evidence of objective memory impairment, no functional impairment, and ability to live independently. All subjects had Clinical Dementia Rating scores of 0.5, consistent with aMCI and at least a high school education. Subjects were free of any other psychiatric or neurological disease. Cognitive tests included (1) the minimental state exam (MMSE) for global cognition, (2) the logical memory I and II subscales of the Wechsler memory scale-revised for immediate and delayed recall, and (3) the Alzheimer's disease assessment scale. Executive functioning was assessed with the Stroop Interference Task and Trails A & B tests. Two subjects were unable to complete the Stroop task due to color blindness. The study was approved by the University of Washington Institutional Review Board and all participants provided informed consent.

Amnestic MCI is generally assumed to be a transition period between normal aging and Alzheimer dementia. Although the diagnostic criteria have been debated, aMCI is commonly defined as a specific impairment in memory that is greater than one would expect for age with a relative normal sparing of other cognitive abilities including attention, judgment, reasoning, and perception. Our sample of aMCI participants included MMSE scores ranging from 21–30, including three individuals with scores of less than 25, scores that are typically considered outside of the aMCI range (Table 1). However, all subjects had CDR scores of 0.5 and a mean ADAS-cog total score of 9.84 ± 1.3 SD, which falls in range with previous published studies of aMCI individuals (c.f. [30]: 11.3 ± 4.4 SD) and is lower than individuals meeting probable AD diagnostic criteria (18.0 ± 6.0). Thus, while most of the participants meet the Petersen criteria for aMCI [31] a few individuals may have been at or near the early transition period of AD at the time of scanning.

### 2.2. MRI Acquisition

Scanning procedures were conducted on a Philips 3.0 T Achieva scanner using an 8-channel SENSE head coil. The scanning protocol included a Magnetization prepared rapid gradient echo (MPRAGE) high-resolution T1 sequence (repetition time (TR)/echo time (TE)/flip angle: 6.5 milliseconds (ms)/3 ms/8°; matrix size of 256 × 256 and with 170 sagittally collected slices and a slice thickness of 1 mm), which was reconstructed in real-time to guide MRS voxel placement and offline hippocampal volumetric calculation.

### 2.3. 1H J-Resolved Magnetic Resonance Spectroscopy

#### 2.3.1. Spectral Estimation of Lactate

**Figure 1:**
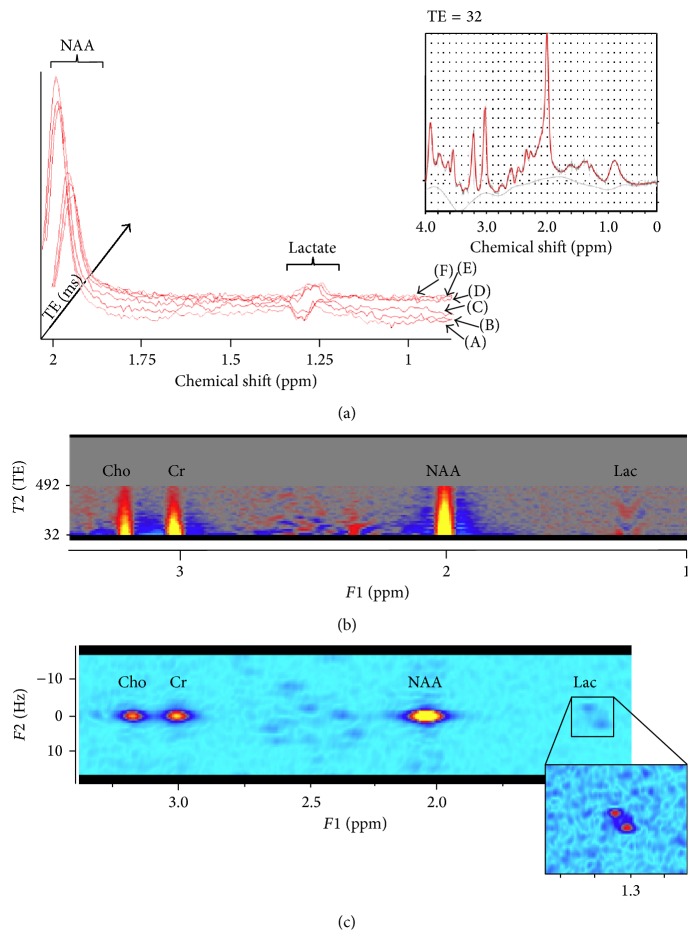
*1H J-resolved MRS lac imaging and analyses*. (a) 3D-plot of averaged, phased proton spectra (filtered for viewing from 2 to 0.8 ppm) for 6 selected echo times (A: 72 ms, B: 92 ms, C: 112 ms, D: 232 ms, E: 252 ms, and F: 272 ms) reveals the inversion of the lac doublet located at 1.3 ppm. The full 1H spectrum at echo time of 32 ms (inset) was used to fit the classic 1H short echo NAA metabolite for absolute quantification using LCmodel. (b) Fourier transformation of the standard acquisition dimension (i.e., chemical shift) converts each FID to a frequency spectrum (F1). The NAA signal for each echo time for each individual was used for quality control purposes and for phase stabilization. No tilt correction was applied in order to inspect possible corruption by T2 noise. (c) A second Fourier transform was applied to the data along the incremented echo time (spectral dimension or F2), converting the oscillating phases of each of the coupled metabolite peaks to their respective frequency components. This contour plot of the 2D spectra, along with projections on the F1 and F2 axes over the chemical shift frequency range was used to localize, isolate, and quantify lactate at the chemical shift frequency of 1.33 ppm at J-resolved frequency of 7.5 Hz.

For each subject, a 2-dimensional J-rMRS acquisition sequence (Figure 1) was acquired from a 4 × 4 × 4 cm voxel that was placed within the PPC, bilaterally. Because lac typically exists in low concentrations in normal physiological conditions, a large voxel size was selected to improve signal to noise. The parameters used for the 2D J-rMRS sequence were PRESS single voxel pulse sequence, TE-steps = 24 (32–492 ms, 20 ms increments, only the 2nd echo intervals were incremented surrounding the 2nd 180 leaving the 1st echo intervals fixed surrounding the 1st 180 in the PRESS pulse sequence), TR = 2 s, spectral bandwidth = 2000 Hz, complex time points = 2048, NEX = 12, and total scan duration = 9.6 min. A spectrum was also acquired with and without water suppression (TE 32 ms) for processing with LCmodel.

### 2.4. Resting State fMRI

An 8-minute resting state, echo planar fMRI sequence (TR/TE/FA: 2000/21/90°, 68 axially oriented slices; matrix size 64 × 64) was acquired to quantify functional connectivity across subjects. Five “dummy” volumes were acquired but excluded from analyses in order to stabilize T1 equilibrium effects. Physiological activity was collected using a custom-made LabView monitoring system with pulse oximetry and a respiratory belt.

## 3. Analysis

### 3.1. MRS Preprocessing

Short echo metabolites fits and quantification were accomplished using LCmodel. Metabolite uncertainties were expressed as % standard deviations (SD). Percent SD, FWHM (full width at half maximum, an estimate of peak width), and S/N (signal-to-noise ratio) were used as determinants of spectrum quality. Free-induction decays (FIDs) were input into the software package and were zero-filled to double the points and filtered with a finite discrete convolution to account for field inhomogeneities and eddy currents. During preprocessing the residual water signal was subtracted by using a decomposition-fitting algorithm. FIDs were then zero- and first-order phase corrected and smoothed using a 1.1 Hz exponential dampening filter. A nonlinear, least squares analysis estimated the metabolite concentrations and their uncertainties.

### 3.2. MRS Analysis

The raw 2D MRS spectra (2048 complex points along the t1 dimension and 24 points along the t2 dimension) were processed offline. One of the spectra (at TE 32 ms, see Figure 1(a) inset) was isolated and used to calculate absolute concentrations of NAA under the t1 dimension. NAA spectral fits were accomplished with LCmodel using standard procedures. Absolute concentrations of NAA were obtained by scaling the in vivo spectrum to the unsuppressed endogenous water peak and are reported in units that approximate millimolar (mM) concentrations.

Partial volume corrections were made by calculating the fraction of cerebral spinal fluid (CSF) for each voxel. CSF, gray matter (GM) and white matter (WM) volume measurements of the PPC voxel were obtained through intensity estimates using the FANTASM MIPAV algorithm (http://mipav.cit.nih.gov/pubwiki/index.php/Main_Page). Metabolite values were then normalized to correct CSF partial volume effects to = 100% brain tissue using: *C* = *C*_*o*_∗(1/(1 − FCSF)) where *C* = concentration, *C*_*o*_ = metabolite concentration from LCmodel output, and FCSF = estimated fraction CSF in order.

The 2D MRS analyses were achieved through in-house software and included a filtered (Sine-bell) Fourier transformation (FT) of the standard chemical shift dimension (t1), which was used to convert each FID to a frequency spectrum (f1). A second FT was then applied to the data along the incremented echo time or spectral dimension (t2), converting the oscillating phases of each of the coupled metabolite peaks to their respective frequency components (f2). After the 2D FT, spectra were converted to power estimates by adding the real squared to the imaginary squared output from the 2D FT. No phasing was necessary for the final FT output due to phasings/frequency shift corrections after the 1st FT to correct for phase imperfections/instabilities of the scanner. The 2D spectra were then plotted as contour plots (Figures 1(b) and 1(c)), revealing the J-coupled frequency of lac at 1.3 ppm as an off midline frequency. The volume under the 2D spectrum (i.e., area under the curve) was determined for lac using manual peak picking on contours and calculated as a ratio to NAA. To estimate absolute concentration, lac values were then multiplied by the LCmodel output of CSF adjusted NAA concentrations.

### 3.3. Hippocampal Volumes

MPRAGE scans were reconstructed into a 1 × 1 × 1 mm 3D volume. Hippocampal volumes were determined on the MPRAGE through a combination of semiautomatic segmentation and extraction using FMRIB's Integrated Registration and Segmentation Tool (FIRST) in FMRIB's Software Library (FSL) version 4.1.5. Following FIRST extraction of hippocampal masks, an expert, blind rater (EHA) inspected and made manual adjustments when necessary so each mask conformed to established rules and boundaries [32].

### 3.4. Intracranial Volumes (ICV)

The ICV for each individual was calculated using a stereologic Cavalieri technique [33] implemented through MEASURE software. Volumes were calculated from a 3D grid of points overlaying the intracranial cavity. This grid was manually identified for each subject by a single rater (KEW). The selected area included all points falling within the cerebrum, cerebellum, sulcal and ventricular CSF, and brainstem superior to the foramen magnum. Volumes were then estimated using the Gundersen formula [34].

### 3.5. Resting State fMRI (rsfMRI)

At the individual level, preprocessing steps to minimize nonneural sources of noise included skull stripping, motion correction with FSL MCFLIRT using a six-parameter rigid body correction and a trilinear interpolation algorithm, with alignment occurring to the middle volume, physiological correction using RETROICOR (implemented through AFNI image processing software [35]), spatial smoothing using 8 mm full width half maximum (FWHM) Gaussian kernel and a low pass temporal filter of 0.1 Hz., grand-mean intensity normalization and linear drift removal. Note that a hardware malfunction prevented the acquisition of physiological activity in one subject. To control physiological noise for this subject within the fMRI data, we calculated and regressed mean resting state white matter and CSF signal prior to spatial smoothing and temporal filtering.

Posterior cingulate seed point functional connectivity analysis was conducted using FSL FEAT toolbox (http://www.fmrib.ox.ac.uk/fsl/feat), importing the coordinates from a previous FDG-PET study of glucose metabolism of preclinical AD as the seed point location [26]. Individual posterior cingulate functional connectivity maps were generated across the whole brain. For each individual, the MNI template brain was initially registered into native fMRI space and the mean time course from a rectangular seed point (4 × 1 × 1 cm) was extracted. The 6 covariates (the 6 motion parameters) were added into a multiple regression analysis as nuisance variables of no-interest. The seed point time course was then entered into whole-brain, voxelwise general linear model analysis using a fixed-effects model and normalizing at the individual level by converting estimates into *Z*-scores.

### 3.6. Statistical Analyses

Statistical relationships between metabolite concentrations and clinical cognitive variables were investigated using parametric partial correlations correcting for numerous known confounds including age and CSF and WM volumes within the MRS voxel. Statistical significance across regressions was determined at a standard alpha level (*P* < 0.05). We also computed significant relationships across rsfMRI PPC functional connectivity and PPC lac concentrations at the group level. Individual seed point rsfMRI time series were orthogonalized and analyses were carried out using a mixed-effects model (FLAME) incorporating PPC MRS lac concentrations as a covariate of interest. Corrections for multiple comparisons were carried out at the cluster level using Gaussian random field theory (minimum voxel *Z*-score, 2.3; cluster significance, *P* < 0.05, corrected).

## 4. Results

From our 2D spectral editing techniques, we observed the characteristic lac doublet signature extracted from the PPC in all 15 aMCI participants (for representative see Figures 1(a)–1(c)). Lac quantification was accomplished using a combination of 2D spectral transforms and LCmodel metabolite estimation. Across subjects these values ranged from 0.16 millimolar (mM) to 0.98 mM (mean 0.587 mM, see Table 1) which are in line with previous efforts to quantify lac under normoxic conditions, localized to superior frontoparietal cortex estimated around 0.5 mM [36], and are in good agreement with quantification estimates from more contemporary findings [37].

### 4.1. PPC Lactate Is Specifically Associated with Memory Capacity in aMCI

**Figure 2:**
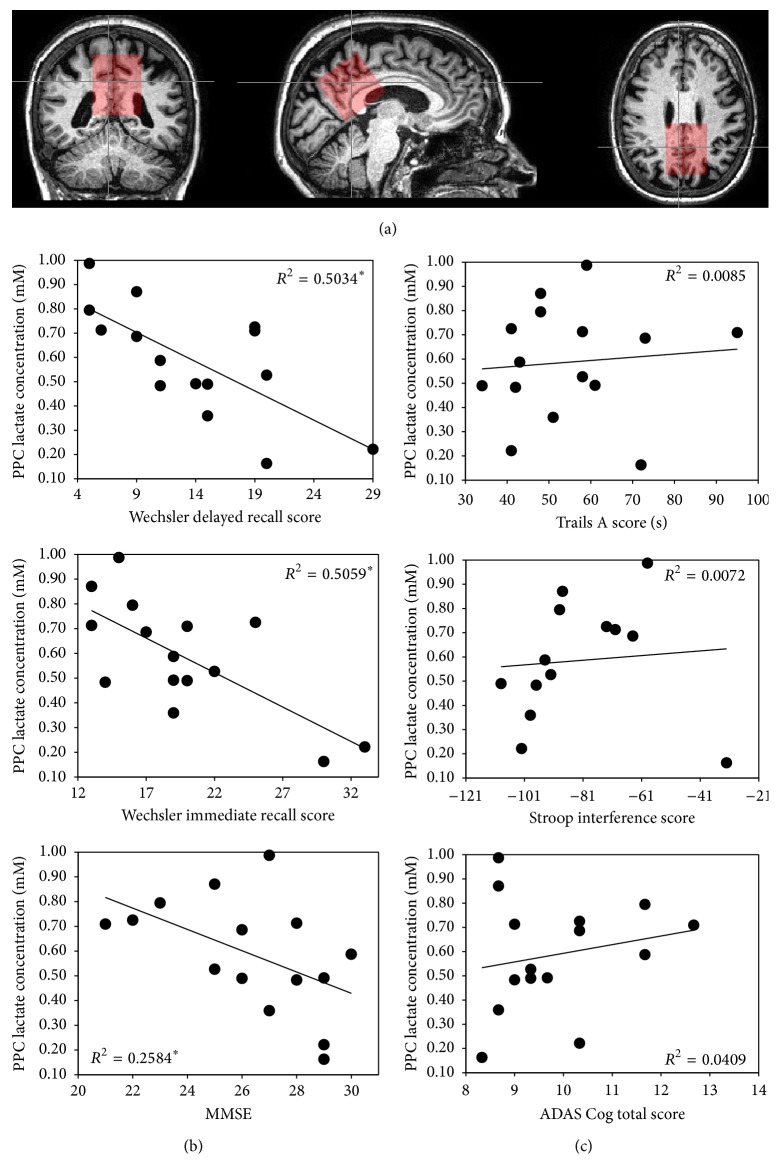
*Posterior cingulate and precuneus (PPC) MRS voxel and lac specific associations.* (a) During scanning, a 4 × 4 × 4 cm voxel (shown in red) was placed bilaterally, centered on medial parietal lobe, covering the posterior cingulate, the precuneus, in most individuals the retrosplenial cortex and in a few individuals the isthmus. A large voxel size is required to provide high lac signal to noise. To ensure consistent placement across subjects, the voxel was centered along the midline, placed just anterior to the parietoccipital fissure and angled in-line with cerebellar tentorium. The voxel was toggled left-to-right to avoid as much ventricular CSF as possible. PPC lac concentrations (*y*-axes) were correlated with performance on various neuropsychological tests including (b) memory based tasks and (c) non-memory specific tasks.  ^*^Correlations were significant at *P* < 0.05 (partial correlations) after correcting for age, GM, and CSF. No correlations on non-memory tasks were statistically significant.

Across subjects, lac concentration was significantly negatively correlated with memory performance on the WMS-R immediate and delayed recall subscales and the MMSE (Figure 2(b)). Negative correlations remained significant after accounting for gray matter (GM) and cerebrospinal fluid (CSF) volumes from the PPC voxel, as well as age (i.e., partial correlations adjusting for GM, CSF, and age; lac and 1, delayed recall: *r* = −0.622, *P* = 0.031, 2, immediate recall: *r* = −0.608, *P* = 0.036 on the WMS, and 3, MMSE: *r* = −0.600, *P* = 0.039). However, no significant associations were observed between lac levels and neuropsychological tasks not specifically tapping memory-related processes (i.e., *P* > 0.05 for Stroop task; Trails A&B; ADAS-Cog total score; Figure 2(c)).

### 4.2. PPC Lactate Is Associated with aMCI Brain Pathology

**Figure 3:**
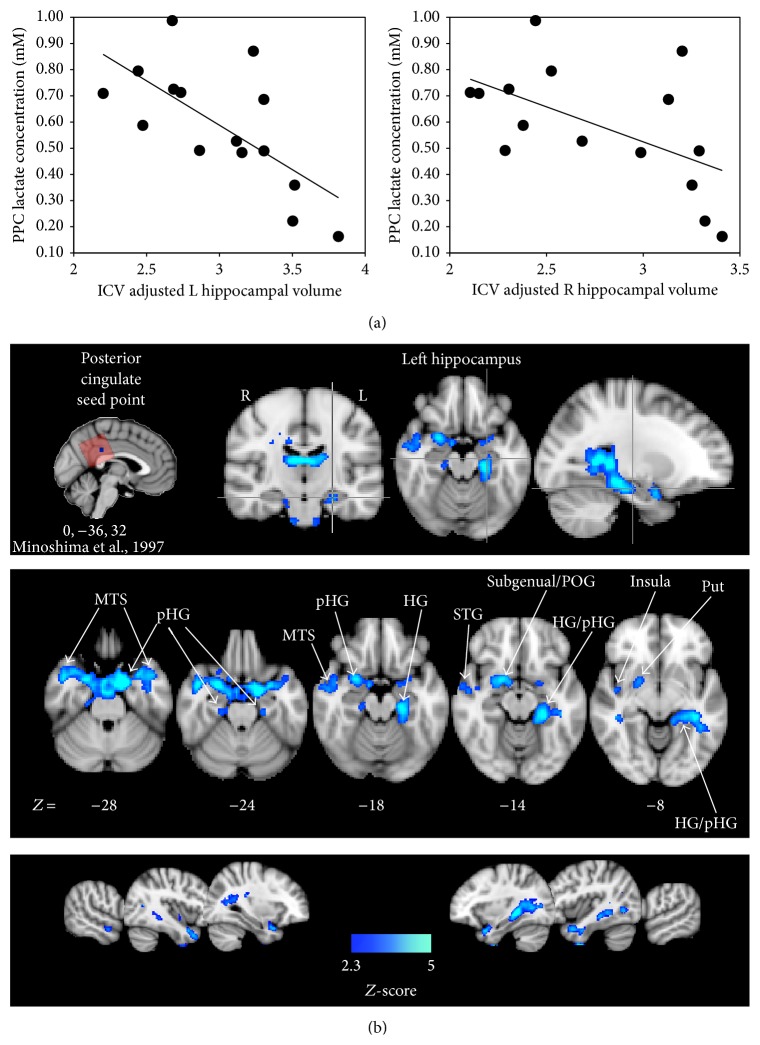
*PPC lac correlations and brain pathology in aMCI.* (a) PPC lac negatively correlates with ICV adjusted L (left) and R (right) hippocampal volumes. (b) Regions of posterior cingulate resting state fMRI functional connectivity that negatively correlate with PPC lac concentrations across aMCI. The PPC seed point (upper left inset) was selected with reference to the site of maximum FDG-PET impairment in a group of preclinical probable AD patients [26] and was contained within MRS voxel locations in all subjects (red transparent box). *Z*-statistics were calculated utilizing a Gaussian random field theory, thresholded using clusters determined by *Z* > 2.3 and a (corrected) cluster significance threshold of *P* = 0.05. Blue voxels showing significant negative correlations between the strength of functional connectivity and PPC lac concentrations, displayed on the MNI152 template brain and in radiological convention, were generally located within the temporal lobe including medial temporal loci (e.g., left hippocampus in upper left green crosshairs) and various subcortical structures (see Table 2). Coordinates (*X*, *Y*, and *Z*) are in millimeters in MNI space. MTG: middle temporal gyrus. pHG: parahippocampal gyrus. STS: superior temporal sulcus. HG: hippocampal gyrus. STG: superior temporal gyrus. POG: posterior orbital gyrus. Put: putamen.

Given the highly specific nature of the lac-memory associations, we sought to determine if lac concentrations across participants were associated with additional indices of disease progression. We first determined left and right hippocampal volumes from the high-resolution T1 MPRAGE. After adjusting for intracranial volumes (ICV), both the left and right hippocampal volumes were negatively correlated with PPC lac levels (Figure 3(a)). Partial correlations adjusting for PPC CSF, GM, and age were not significant (*P* > 0.05). However after removing CSF from the regression model, the correlations were significant (*r* = 0.589, *P* = 0.032 for the left hippocampus, and *r* = 0.432, *P* = 0.044, for the right hippocampus), suggesting an interaction between PPC CSF concentration, lac concentrations, and hippocampal volume.

Resting state fMRI scans were acquired to examine the association between PPC lac and posterior cingulate functional connectivity. After correcting for physiological activity (i.e., cardiac cycle and respiration), whole-brain, voxelwise resting state time courses were extracted from a seed point placed at the site of maximum decrease of FDG-PET consumption across probable preclinical AD patients in a previous study [26], a location which overlapped with the placement of the MRS voxel (Figure 3(b)) in all subjects. Lac concentrations were then entered into a group-level analysis as a covariate of interest using a mixed-effects model and correcting for multiple comparisons at the cluster level using a Gaussian random field theory (minimum voxel *Z*-score, 2.3; cluster significance, *P* = 0.05, corrected). Significant negative correlations between PPC functional connectivity and lac concentrations across the group were observed throughout the temporal lobe (Figure 3(b) and Table 2). In particular, the left hippocampus (Figure 3(b), left side) as well as the parahippocampal gyrus bilaterally had a substantial number of voxels showing significant negative correlations between the degree of posterior cingulate functional connectivity and PPC lac concentrations.

## 5. Discussion

We observed that J-rMRS quantification of lactate, the by-product of the anaerobic metabolic process of glycolysis, within a cortical node of the system for memory retrieval is significantly associated with common cognitive and imaging markers of disease progression (e.g., delayed memory function, hippocampal volume, and functional connectivity) in a cohort of aMCI individuals. Previous MRS efforts have failed to detect differences or a lac resonance altogether [21, 22]. This discrepancy is likely due to fact that these studies did not account for the background of overlapping lipid and macromolecule contamination that occurs at the same chemical shift frequency exhibited by lac. J-rMRS is a more sensitive means for isolating lac than traditional short-TE MRS methods because it utilizes the J-coupling properties of lac, physical properties lacking in macromolecules and lipids.

Collectively, we propose J-rMRS as a complementary and/or alternative metabolic biomarker to track the metabolic status in aMCI and possibly AD. MRS is completely noninvasive. Quantification of metabolically active biomolecules with MRS requires minimal time commitment while providing in vivo access to disease-sensitive tissue of interest. Consequently MRS is appropriate and routinely used in clinical diagnostics for metabolic disorders [19, 38]. Relative to FDG PET, J-rMRS would likely provide a preferable means of tracking metabolic decline when repeated measurements over short time periods are necessary, for example in clinical trials targeting metabolic function and examining therapeutic response [12, 39].

### 5.1. PPC Lac Concentrations as a Function of Disease Severity

PPC lac levels were only significantly associated with cognitive tests of memory (including the WMS-R delayed and immediate recall, Figure 2(b)). Critical for prediction of AD conversion is the observation that aMCI individuals presenting with the most impacted delayed recall are at greatest risk for developing AD [40]. The observed negative association between lac and memory function indicates that higher levels of PPC lac are related to poorer memory retention, with nearly 50% of the variance in WMS-R subscales performance attributed to PPC lac concentrations (Figure 2(b)). Our explicit statistical adjustments for age, CSF concentrations of lac [27], and GM volume within the PPC voxel (c.f. [41]) give further justification in interpreting the finding as a disease-specific effect. This point is further supported by the significant negative relationship between PPC lac concentration and MMSE scores, a traditional clinical assessment tool. Although follow-up studies will be needed to determine whether individuals with higher PPC lac concentrations are more likely to convert to AD, these observations do suggest elevated levels of PPC lac are linked to aMCI behavioral impairment and by extension are likely related to disease-specific brain pathology.

We tested this assumption by examining the association of PPC lac with two common markers aMCI/AD brain pathology: hippocampal volume and posterior cingulate functional connectivity (Figure 3). The negative correlations between PPC lac levels and hippocampal volume (Figure 3(a)) parallel PPC FDG-PET observations and support the notion that hippocampal atrophy is associated with PPC metabolic function in AD [3]. It is worth commenting on the observation that PPC CSF concentrations accounted for a portion of variance in hippocampal volumes (but not the PPC lac, memory associations). This suggests the possibility of measureable lac concentrations within CSF, a notion that has yet to be explored in aMCI (as far as we are aware), and possibly contributing to the overall PPC lac concentrations across participants. However PPC lac was still significantly correlated with memory ability across the sample even after controlling CSF concentration within the PPC voxel. This would indicate that the concentration of PPC lac, above and beyond CSF levels, is significantly associated with aMCI behavioral impairment, a cognitive process that is in part a function of hippocampal-PPC interactions [5, 6].

In an attempt to reconcile spatial differences between the location of the PPC MRS voxel and the FDG-PET discrepancies in aMCI/AD, we imported the coordinates corresponding to the greatest reduction in FDG-PET utilization from a previous PET study of probable prodromal AD [26] as a seed point location for the resting state time point analysis. Individuals with higher concentrations of PPC lac had greater reductions of functional connectivity between the PPC and numerous cortical and subcortical regions, including the left hippocampus, parahippocampal gyrus, and middle temporal gyrus (Figure 3(b) and Table 2), a spatial topography that parallels resting state functional connectivity maps extracted from the posterior cingulate in neurologically normal individuals [42]. Previous imaging efforts have shown that the strength of connectivity between the hippocampus and the PPC predicts memory capacity in cognitively intact older adults [5] and that this connectivity is degraded in aMCI [43] and AD [44]. Here our functional connectivity observations support the hypothesis that PPC lac concentration is in part attributed to reduced hippocampal input [41].

The negative relationships between PPC lac and memory function, hippocampal volume, and posterior cingulate functional connectivity indicates that individuals presenting with a greater progression of disease state have increased concentrations of PPC lac. Such characteristics have been shown to predict (with varying degrees of success) which aMCI individuals are most likely to convert to AD [40, 44–46]. Thus, it is feasible that PPC lac concentration may also be a marker of conversion. Because PPC lac-behavioral associations were independent of age, this line of reasoning would posit a critical concentration threshold of PPC lac that would distinguish normal aging from aMCI from AD.

### 5.2. Mechanisms of Lac Production and AD Pathology

Brain lac is produced from the conversion of glucose through glycolysis occurring to a large degree within the cytosol of astrocytes [16]. The byproduct of glycolysis is pyruvate. Classic models of brain metabolism suggest that under normoxic conditions pyruvate enters mitochondria and the oxidative TCA cycle and electron transport chain yielding high levels of ATP. However, in situations when metabolic demands exceed oxygen supply, pyruvate is alternatively catalyzed into lac via the enzymatic activity of lactate dehydrogenase. This combined process, which is referred to as anaerobic glycolysis, yields significantly lower but more rapid amounts of ATP relative to respiration and oxidative phosphorylation [47]. Sustained periods of low oxygen concentrations result in continued production of lac and eventual lactic acidosis. While some studies have shown that concentrations of brain lac can rise as a result of continuous neural stimulation [48], elevated brain lac is generally considered to reflect pathology induced from hypoxia or ischemia [49] or other metabolic crises [50]. However, in certain clinical situations (e.g., congenital mitochondrial disorders) in which mitochondrial function is compromised and cannot sustain ATP levels needed to fuel cellular processes [15], energy requirements are met (at least initially) through a compensatory upregulation of anaerobic glycolysis [17] which leads to elevated lac concentrations [16].

Based upon quantitative reports of metabolic pathology, similar physiological pressures are at play in aMCI all of which could lead to elevated lac in aMCI individuals further along across the disease progression. The conventional interpretation would predict that progressive mitochondrial dysfunction and/or heightened hypoperfusion-induced hypoxia routinely noted throughout the AD spectrum [10, 51] would lead to increases in anaerobic glycolysis, a mitochondrial-independent process as compensation for declining cerebrovascular function/mitochondrial ATP output and accumulating lac concentrations [52].

However, this hypothesis would need to be reconciled with the known reductions of glucose metabolism in the PPC [1, 26, 41] since the supportive fuel for anaerobic glycolysis is glucose. That is, the downregulation of PPC glucose consumption commonly noted in aMCI/AD pathology would in fact, contrary to our observations, predict decreased levels of lac in individuals with more severe pathology. One leading notion underlying FDG-PET impairments in aMCI and AD, particularly within the PPC, is a slow but gradual reduction of energy requirements stemming from damage to the cellular constituents (e.g., synapses) with high metabolic demand [53]. Abnormal mitochondrial function in AD results in an overproduction of reactive oxygen species, increased oxidative stress, and damage [13]. As a consequence, neuronal cell types with higher concentrations of mitochondria, (particularly within axons and synaptic terminals) relative to glial cells are subject to greater levels of AD-induced metabolic impairment, increased oxidative stress, and a greater likelihood of cell damage and death. Therefore, it is conceivable that while neurons and synapses, the tissue substrates likely responsible for driving FDG-PET reductions [54], suffer a greater burden of AD-induced tissue damage, glial cells including astrocytes remain relatively spared (i.e., degraded from AD pathogenesis). Decreasing synaptic function and neuron numbers would reduce net glucose requirements, while a simultaneous response to astrocytic mitochondrial dysfunction would elevate anaerobic glycolysis ultimately driving up lac concentrations. In support of such a metabolic scenario, an in vitro study observed that cultured astrocytes stimulated with a toxic form of the ABeta peptide produced significantly more lac relative to astrocytes that were bathed in a control peptide [55]. Possible additional (and by no means mutually exclusive) mechanisms include the astrocytic response seen during severe hypoxia or ischemia where delivery of brain glucose (and oxygen) is toxically reduced. In MCI and AD, chronic reductions in PPC glucose may result in utilization of astrocytically stored glycogen, a molecule that can be consumed as an alternative energy source to power anaerobic glycolysis and thus raise lac concentrations [56]. Additionally, Bubber and colleagues observed a negative correlation between pyruvate dehydrogenase complex activity in AD postmortem brain tissue and CDR scores taken just prior to autopsy [57]. The pyruvate dehydrogenase complex, a complex consisting of three enzymes housed within the mitochondrial matrix, is responsible in part for converting pyruvate into acetyl-CoA, an enzyme used in the TCA cycle and electron transport chain (i.e., cellular respiration). If this complex is degraded, pyruvate is alternatively catalyzed into lac via lactate dehydrogenase, a core characteristic of pyruvate dehydrogenase complex deficiency (PDCD) syndrome [58].

### 5.3. Applications of J-rMRS Lac as a Metabolic Biomarker in aMCI

Numerous pharmacological and biobehavioral efforts are underway that aim to mollify and normalize metabolic instabilities in at-risk populations [12, 13, 51]. FDG-PET is expected to play a significant role in these studies as a means of tracking cerebral metabolic function. An ideal biomarker of disease progression isolates the physiological process being targeted by a therapeutic. This is a critical factor for assessing therapeutic response as it maximizes the ability to “see” preservations and/or improvements at the physiological level while minimizing the need to rely on functional/behavioral outcomes [39]. Mitochondrial physiology is currently one of the main avenues of investigation for the pharmacological restoration of metabolism in AD [13, 51]. Indeed some therapeutic success has been noted both in vitro and cognitive restoration when applying a mitochondrial-targeted antioxidant to a transgenic mouse model of AD [12]. Most metabolic biomarker efforts to date have either pursued PET imaging or measurements of various peripheral or central metabolically active molecules from the blood or CSF, respectively. However, to anticipate similar success within human clinical trials, a biomarker capable of specifically tracking (a) mitochondrial function or (b) a physiological process that reflects mitochondrial function from the tissue of interest is required. An imaging-based biomarker that is specifically sensitive to mitochondrial dysfunction within specific disease-sensitive cortical regions has not yet been published [59]. At a systems level, FDG-PET reductions in aMCI are assumed to reflect a global loss of tissue substrate in the brain, resulting from both a downstream effect stemming from hippocampal neuronal death leading to subsequent axonal withdrawal and synapse loss (diaschisis) as well as local neuronal and gray matter (GM) atrophy [1, 41]. Altogether, we propose that tracking lac concentrations from AD-sensitive brain regions such as the PPC will provide greater physiology specificity for monitoring the effects of mitochondrial uncoupling and cerebrovascular hypoperfusion across the disease spectrum.

### 5.4. Caveats and Limitations

We show that PPC lac is significantly associated with common aMCI markers of disease progression. Despite the overwhelmingly strong correlations, we cannot specifically conclude that PPC lac is elevated in aMCI until statistical comparisons can be made against age-matched controls presenting with normal memory and cognitive function. Rather we argue that PPC lac provides a means of noninvasively tracking the metabolic status of individuals presenting with significant memory complications. Moreover, we cannot rule out the possibility of elevated lac (as a function of disease pathology) as a more global phenomenon due to the fact that lac concentrations were not quantified from a control region. Given that FDG-PET abnormalities (although generally not uniform across aMCI) have been observed within other association cortices [1], it would not be surprising if lac concentrations are elevated in aMCI in additional cortex, for example, across the DMN and including the medial temporal lobe [4]. Tracking changes in lac over time within subjects will be specifically critical for validating the hypothesis of PPC lac as a biomarker of metabolic dysfunction.

### 5.5. Conclusions

Here we provide preliminary evidence that PPC lac quantified with J-rMRS is negatively associated with markers of disease progression in aMCI, a commonly assumed prodrome of AD. Based on lac imaging of mitochondrial and metabolic disorders, we propose that the well-established alterations in normal metabolic physiology that are linked to disease progression (i.e., mitochondrial impairment and/or cerebrovascular complications leading to hypoxia) likely give rise to exacerbated anaerobic glycolysis and detectable brain lac in aMCI. Accordingly, these relationships suggest that our lac concentrations likely reflect the metabolic status of the PPC, brain tissue that is exceptionally vulnerable to AD pathology. Future studies will need to combine FDG-PET and J-rMRS to determine if PPC lac is associated with the typical glucose consumption deficits. As a result of the completely noninvasive nature of MR imaging, J-rMRS provides a viable and likely preferable means of tracking metabolic function and dysfunction across multiple time points throughout the AD continuum. A link between PPC FDG-PET levels and lac concentrations may suggest the possibility that J-rMRS can augment or potentially supplant the metabolic monitoring capacities of PET. Moreover, future studies will need to link specific markers of mitochondrial dysfunction and/or cerebrovascular impairment with fluctuations in lac concentration across aMCI individuals. If quantitative studies of mitochondrial and cerebrovascular function are indeed tied lac concentrations, our results suggest an in vivo means of interrogating a metabolic process that is the current focus of a number of therapeutic trials [12, 51]. A biomarker providing a greater specificity for the therapeutically targeted pathophysiological process, together with cognitive restoration, will be critical factors establishing direct disease-modifying effects [39].

## Figures and Tables

**Table 1:** Subject demographics, clinical assessment, and lactate MRS outcomes.

Subject	Gender	Age	Handedness^*^	MMSE	ADAS-Cog^$^	PPC MRS voxel			
						Lactate (mM)	Proportion tissue types		
							GM	WM	CSF
MCI1	M	89	Left	23	11.67	0.795	0.34	0.37	0.29
MCI2	F	94	Right	28	9	0.713	0.37	0.33	0.3
MCI3	M	86	Right	21	12.67	0.709	0.31	0.41	0.28
MCI4	F	82	Right	30	11.67	0.587	0.35	0.37	0.27
MCI5	F	87	Right	22	10.33	0.725	0.3	0.46	0.24
MCI6	F	86	Right	26	9.33	0.490	0.35	0.4	0.25
MCI7	F	83	Right	25	8.67	0.871	0.33	0.4	0.28
MCI8	M	79	Right	26	10.33	0.686	0.4	0.41	0.19
MCI9	F	80	Right	29	10.33	0.222	0.36	0.48	0.16
MCI10	F	87	Right	29	8.33	0.163	0.34	0.4	0.25
MCI11	F	82	Right	25	9.33	0.527	0.36	0.39	0.21
MCI12	F	85	Right	28	9	0.483	0.39	0.4	0.21
MCI13	F	82	Right	27	8.67	0.359	0.33	0.46	0.21
MCI14	M	88	Right	27	8.67	0.987	0.35	0.38	0.27
MCI15	M	93	Right	29	9.67	0.491	0.34	0.38	0.26

^*^Based on self-report.

MMSE: minimental state examination.

^$^Total (combined) score.

GM: grey matter; WM: white matter; CSF: cerebral spinal fluid.

**Table 2:** Regions of peak *Z*-scores of voxels showing a significant negative correlation between PPC lac concentration and posterior cingulate functional connectivity.

Region	*Z*-score	Hemisphere	MNI coordinates (mm)			BA
			*x*	*y*	*z*	
Posterior cingulate	7.63	R	16	−40	34	31
Lateral occipital cortex/angular gyrus	7.23	L	−38	−68	16	39
Lateral occipital cortex/angular gyrus	6.93	R	40	−60	12	39
Anterior parahippocampal gyrus	6.92	L	−16	0	−26	34
Hippocampus/parahippocampal gyrus	6.77	L	−26	−38	2	36
Thalamus	6.35	R	12	−22	14	N/A
Inferior temporal gyrus/temporal pole	6.08	L	−36	0	−52	20
Superior temporal sulcus	5.47	R	46	4	−26	22
Hippocampus	5.25	L	−22	−22	−20	N/A
Anterior parahippocampal gyrus	5.12	R	22	2	−26	34
Posterior orbital gyrus/subgenual/putamen	4.94	R	20	4	−14	25
Insular cortex	3.36	R	42	−6	−6	13

BA: Brodmann area.

## References

[1] Mosconi L., Pupi A., de Leon M. J. (2008). Brain glucose hypometabolism and oxidative stress in preclinical Alzheimer's disease. *Annals of the New York Academy of Sciences*.

[2] Chételat G., Desgranges B., de la Sayette V. (2003). Dissociating atrophy and hypometabolism impact on episodic memory in mild cognitive impairment. *Brain*.

[3] Yamaguchi S., Meguro K., Itoh M. (1997). Decreased cortical glucose metabolism correlates with hippocampal atrophy in Alzheimer's disease as shown by MRI and PET. *Journal of Neurology, Neurosurgery and Psychiatry*.

[4] Beason-Held L. L. (2011). Dementia and the default mode. *Current Alzheimer Research*.

[5] Wang L., LaViolette P., O'Keefe K. (2010). Intrinsic connectivity between the hippocampus and posteromedial cortex predicts memory performance in cognitively intact older individuals. *NeuroImage*.

[6] Sestieri C., Corbetta M., Romani G. L., Shulman G. L. (2011). Episodic memory retrieval, parietal cortex, and the default mode network: functional and topographic analyses. *Journal of Neuroscience*.

[7] Reiman E. M. (2011). Fluorodeoxyglucose positron emission tomography: emerging roles in the evaluation of putative Alzheimer's disease-modifying treatments. *Neurobiology of Aging*.

[8] Marchesi V. T. (2011). Alzheimer's dementia begins as a disease of small blood vessels, damaged by oxidative-induced inflammation and dysregulated amyloid metabolism: implications for early detection and therapy. *The FASEB Journal*.

[9] Lin A.-L., Rothman D. L. (2014). What have novel imaging techniques revealed about metabolism in the aging brain?. *Future Neurology*.

[10] Iadecola C. (2004). Neurovascular regulation in the normal brain and in Alzheimer's disease. *Nature Reviews Neuroscience*.

[11] Dai W., Lopez O. L., Carmichael O. T., Becker J. T., Kuller L. H., Gach H. M. (2009). Mild cognitive impairment and Alzheimer disease: patterns of altered cerebral blood flow at MR imaging. *Radiology*.

[12] Mcmanus M. J., Murphy M. P., Franklin J. L. (2011). The mitochondria-targeted antioxidant mitoq prevents loss of spatial memory retention and early neuropathology in a transgenic mouse model of Alzheimer's disease. *Journal of Neuroscience*.

[13] Reddy P. H., Tripathi R., Troung Q. (2012). Abnormal mitochondrial dynamics and synaptic degeneration as early events in Alzheimer’s disease: implications to mitochondria-targeted antioxidant therapeutics. *Biochimica et Biophysica Acta*.

[14] Sijens P. E., Smit G. P. A., Rödiger L. A. (2008). MR spectroscopy of the brain in Leigh syndrome. *Brain and Development*.

[15] Saneto R. P., Friedman S. D., Shaw D. W. (2008). Neuroimaging of mitochondrial disease. *Mitochondrion*.

[16] Schurr A. (2006). Lactate: the ultimate cerebral oxidative energy substrate?. *Journal of Cerebral Blood Flow & Metabolism*.

[17] Almeida A., Almeida J., Bolaños J. P., Moncada S. (2001). Different responses of astrocytes and neurons to nitric oxide: the role of glycolytically generated ATP in astrocyte protection. *Proceedings of the National Academy of Sciences of the United States of America*.

[18] Berthet C., Lei H., Thevenet J., Gruetter R., Magistretti P. J., Hirt L. (2009). Neuroprotective role of lactate after cerebral ischemia. *Journal of Cerebral Blood Flow and Metabolism*.

[19] Lin D. D. M., Crawford T. O., Barker P. B. (2003). Proton MR spectroscopy in the diagnostic evaluation of suspected mitochondrial disease. *American Journal of Neuroradiology*.

[20] Cady E. B. (2001). Magnetic resonance spectroscopy in neonatal hypoxic-ishaemic insults. *Child's Nervous System*.

[21] Stoppe G., Bruhn H., Pouwels P. J. W., Hänicke W., Frahm J. (2000). Alzheimer disease: absolute quantification of cerebral metabolites in vivo using localized proton magnetic resonance spectroscopy. *Alzheimer Disease and Associated Disorders*.

[22] Ernst T., Chang L., Melchor R., Mehringer C. M. (1997). Frontotemporal dementia and early Alzheimer disease: differentiation with frontal lobe H-1 MR spectroscopy. *Radiology*.

[23] Govindaraju V., Young K., Maudsley A. A. (2000). Proton NMR chemical shifts and coupling constants for brain metabolites. *NMR in Biomedicine*.

[24] Dager S. R., Corrigan N. M., Richards T. L., Posse S. (2008). Research applications of magnetic resonance spectroscopy to investigate psychiatric disorders. *Topics in Magnetic Resonance Imaging*.

[25] Soares D. P., Law M. (2009). Magnetic resonance spectroscopy of the brain: review of metabolites and clinical applications. *Clinical Radiology*.

[26] Minoshima S., Giordani B., Berent S., Frey K. A., Foster N. L., Kuhl D. E. (1997). Metabolic reduction in the posterior cingulate cortex in very early Alzheimer's disease. *Annals of Neurology*.

[27] Li Y., Chen A. P., Crane J. C., Chang S. M., Vigneron D. B., Nelson S. J. (2007). Three-dimensional J-resolved H-1 magnetic resonance spectroscopic imaging of volunteers and patients with brain tumors at 3T. *Magnetic Resonance in Medicine*.

[28] Thomas M. A., Ryner L. N., Mehta M. P., Turski P. A., Sorenson J. A. (1996). Localized 2D J-resolved 1H MR spectroscopy of human brain tumors in vivo. *Journal of Magnetic Resonance Imaging*.

[29] Petersen R. C., Smith G. E., Waring S. C., Ivnik R. J., Tangalos E. G., Kokmen E. (1999). Mild cognitive impairment: clinical characterization and outcome. *Archives of Neurology*.

[30] Grundman M., Petersen R. C., Ferris S. H. (2004). Mild cognitive impairment can be distinguished from Alzheimer disease and normal aging for clinical trials. *Archives of Neurology*.

[31] Petersen R. C., Doody R., Kurz A., etal (2001). Current concepts in mild cognitive impairment. *Archives of Neurology*.

[32] Honeycutt N. A., Smith P. D., Aylward E. (1998). Mesial temporal lobe measurements on magnetic resonance imaging scans. *Psychiatry Research*.

[33] Barta P. E., Dhingra L., Royall R., Schwartz E. (1997). Improving sterological estimates for the volume of structures identified in three-dimensional arrays of spatial data. *Journal of Neuroscience Methods*.

[34] Gundersen H. J. G., Bagger P., Bendtsen T. F. (1988). The new stereological tools: disector, fractionator, nucleator and point sampled intercepts and their use in pathological research and diagnosis. *APMIS*.

[35] Glover G. H., Li T. Q., Ress D. (2000). Image-based method for retrospective correction of physiological motion effects in fMRI: RETROICOR. *Magnetic Resonance in Medicine*.

[36] Hanstock C. C., Rothman D. L., Prichard J. W., Jue T., Shulman R. G. (1988). Spatially localized 1H NMR spectra of metabolites in the human brain. *Proceedings of the National Academy of Sciences of the United States of America*.

[37] Edden R. A. E., Harris A. D., Murphy K. (2010). Edited MRS is sensitive to changes in lactate concentration during inspiratory hypoxia. *Journal of Magnetic Resonance Imaging*.

[38] Corrigan N. M., Richards T. L., Friedman S. D., Petropoulos H., Dager S. R. (2010). Improving 1H MRSI measurement of cerebral lactate for clinical applications. *Psychiatry Research-Neuroimaging*.

[39] Blennow K. (2010). Biomarkers in Alzheimer's disease drug development. *Nature Medicine*.

[40] Chen P., Ratcliff G., Belle S. H., Cauley J. A., DeKosky S. T., Ganguli M. (2000). Cognitive tests that best discriminate between presymptomatic AD and those who remain nondemented. *Neurology*.

[41] Villain N., Desgranges B., Viader F. (2008). Relationships between hippocampal atrophy, white matter disruption, and gray matter hypometabolism in Alzheimer's disease. *The Journal of Neuroscience*.

[42] Margulies D. S., Vincent J. L., Kelly C. (2009). Precuneus shares intrinsic functional architecture in humans and monkeys. *Proceedings of the National Academy of Sciences of the United States of America*.

[43] Sorg C., Riedl V., Mühlau M. (2007). Selective changes of resting-state networks in individuals at risk for Alzheimer's disease. *Proceedings of the National Academy of Sciences of the United States of America*.

[44] Zhou Y., Dougherty J. H., Hubner K. F., Bai B., Cannon R. L., Hutson R. K. (2008). Abnormal connectivity in the posterior cingulate and hippocampus in early Alzheimer's disease and mild cognitive impairment. *Alzheimer's and Dementia*.

[45] Chételat G., Desgranges B., De la Sayette V., Viader F., Eustache F., Baron J.-C. (2003). Mild cognitive impairment: can FDG-PET predict who is to rapidly convert to Alzheimer's disease?. *Neurology*.

[46] Drzezga A., Lautenschlager N., Siebner H. (2003). Cerebral metabolic changes accompanying conversion of mild cognitive impairment into alzheimer's disease: a PET follow-up study. *European Journal of Nuclear Medicine and Molecular Imaging*.

[47] Chi C. P., Roberts E. L. (2003). Energy substrates for neurons during neural activity: a critical review of the astrocyte-neuron lactate shuttle hypothesis. *Journal of Cerebral Blood Flow and Metabolism*.

[48] Prichard J., Rothman D., Novotny E. (1991). Lactate rise detected by 1H NMR in human visual cortex during physiologic stimulation. *Proceedings of the National Academy of Sciences of the United States of America*.

[49] Shalak L., Perlman J. M. (2004). Hypoxic-ischemic brain injury in the term infant-current concepts. *Early Human Development*.

[50] Sijens P. E., Levendag P. C., Vecht C. J., van Dijk P., Oudkerk M. (1996). 1H MR spectroscopy detection of lipids and lactate in metastatic brain tumors. *NMR in Biomedicine*.

[51] Aliev G., Palacios H. H., Walrafen B., Lipsitt A. E., Obrenovich M. E., Morales L. (2009). Brain mitochondria as a primary target in the development of treatment strategies for Alzheimer disease. *International Journal of Biochemistry and Cell Biology*.

[52] Dienel G. A., Cruz N. F. (2008). Imaging brain activation: simple pictures of complex biology. *Annals of the New York Academy of Sciences*.

[53] Selkoe D. J. (2002). Alzheimer's disease is a synaptic failure. *Science*.

[54] Kadekaro M., Crane A. M., Sokoloff L. (1985). Differential effects of electrical stimulation of sciatic nerve on metabolic activity in spinal cord and dorsal root ganglion in the rat. *Proceedings of the National Academy of Sciences of the United States of America*.

[55] Allaman I., Gavillet M., Bélanger M. (2010). Amyloid-beta aggregates cause alterations of astrocytic metabolic phenotype: impact on neuronal viability. *Journal of Neuroscience*.

[56] Rossi D. J., Brady J. D., Mohr C. (2007). Astrocyte metabolism and signaling during brain ischemia. *Nature Neuroscience*.

[57] Bubber P., Haroutunian V., Fisch G., Blass J. P., Gibson G. E. (2005). Mitochondrial abnormalities in Alzheimer brain: mechanistic implications. *Annals of Neurology*.

[58] Shevell M. I., Matthews P. M., Scriver C. R. (1994). Cerebral dysgenesis and lactic acidemia: an MRI/MRS phenotype associated with pyruvate dehydrogenase deficiency. *Pediatric Neurology*.

[59] Blass J. P. (2008). A new approach to treating Alzheimer's disease. *Annals of the New York Academy of Sciences*.

